# Viral infections in outpatients with medically attended acute respiratory illness during the 2012–2013 influenza season

**DOI:** 10.1186/s12879-015-0806-2

**Published:** 2015-02-22

**Authors:** Richard K Zimmerman, Charles R Rinaldo, Mary Patricia Nowalk, GK Balasubramani, Krissy K Moehling, Arlene Bullotta, Heather F Eng, Jonathan M Raviotta, Theresa M Sax, Stephen Wisniewski

**Affiliations:** Department of Family Medicine, University of Pittsburgh School of Medicine, Pittsburgh, PA USA; Department of Pathology, University of Pittsburgh School of Medicine, Pittsburgh, PA USA; Departments of Infectious Disease and Microbiology, and Epidemiology, University of Pittsburgh Graduate School of Public Health, Pittsburgh, PA USA; Department of Epidemiology, University of Pittsburgh Graduate School of Public Health, Pittsburgh, PA USA

**Keywords:** Influenza, Viral infections, Virus detection, RSV

## Abstract

**Background:**

While it is known that acute respiratory illness (ARI) is caused by an array of viruses, less is known about co-detections and the resultant comparative symptoms and illness burden. This study examined the co-detections, the distribution of viruses, symptoms, and illness burden associated with ARI between December 2012 and March 2013.

**Methods:**

Outpatients with ARI were assayed for presence of 18 viruses using multiplex reverse transcriptase polymerase chain reaction (MRT-PCR) to simultaneously detect multiple viruses.

**Results:**

Among 935 patients, 60% tested positive for a single virus, 9% tested positive for ≥1 virus and 287 (31%) tested negative. Among children (<18 years), the respective distributions were 63%, 14%, and 23%; whereas for younger adults (18–49 years), the distributions were 58%, 8%, and 34% and for older adults (≥50 years) the distributions were 61%, 5%, and 32% (*P* < 0.001). Co-detections were more common in children than older adults (*P* = 0.01), and less frequent in households without children (*P* = 0.003). Most frequently co-detected viruses were coronavirus, respiratory syncytial virus, and influenza A virus. Compared with single viral infections, those with co-detections less frequently reported sore throat (*P* = 0.01), missed fewer days of school (1.1 vs. 2 days; *P* = 0.04), or work (2 vs. 3 days; *P* = 0.03); other measures of illness severity did not vary.

**Conclusions:**

Among outpatients with ARI, 69% of visits were associated with a viral etiology. Co-detections of specific clusters of viruses were observed in 9% of ARI cases particularly in children, were less frequent in households without children, and were less symptomatic (e.g., lower fever) than single infections.

## Background

Each year, hundreds of millions of people are afflicted with viral respiratory tract infections most commonly caused by human adenovirus (ADNO), human coronavirus (CoV), human metapneumovirus (HMPV), human rhinovirus (HRV), influenza virus (influenza), parainfluenza virus (PIV), and respiratory syncytial virus (RSV). Moderate to severe respiratory tract infections may lead patients to seek outpatient medical attention. Yet, only a portion of these medically attended acute respiratory infections (ARI) has been routinely tested to determine their etiology because rapid testing can be expensive and treatment options for viral infections are limited. New assays using multiplex reverse transcriptase polymerase chain reactions (MRT-PCR) are available allowing for relatively rapid detection of multiple virus types and simultaneous comparison of patient characteristics, symptoms, severity of illness and productivity across multiple viruses.

During the 2012–2013 influenza season, the multi-center U.S. Influenza Vaccine Effectiveness (Flu VE) Network, funded by the Centers for Disease Control and Prevention (CDC), conducted a study to determine the effectiveness of the season’s influenza vaccine using singleplex RT-PCR (SRT-PCR) to detect influenza virus. The University of Pittsburgh site of the Flu VE Network also used MRT-PCR.

### Objectives

The purposes of this study were to: 1) examine the distribution of viruses associated with ARI visits during December 2012 through March 2013 in Allegheny County, Pennsylvania, using MRT-PCR; and 2) compare demographic characteristics, symptoms, and consequences of various infections and of co-detections vs. single or no infections. A previous, similar study [1] used the same methodology to examine ARI in 2011–2012. Because the onset, severity and length of the influenza season vary year to year, the present study differs in its epidemiology and its focus on viral co-detections.

## Methods

### Study design

#### Participants

Participants provided informed consent and were enrolled in the University of Pittsburgh’s center for the US Flu VE Network study described previously [2]. The parent study used a test negative case control study design [3,4], where the proportion vaccinated among those who test positive for influenza is compared with the proportion vaccinated among those who test negative. Eligibility criteria included age ≥6 months as of 9/1/2012 and presentation at one of the participating primary care centers for treatment of an upper respiratory illness of ≤7 days duration, with cough, and not taking an influenza antiviral (oseltamivir or zanamivir) before the medical visit. Results from influenza testing were not available soon enough for clinical decision-making with regard to antiviral prescribing. Antiviral medication prescribed as a result of the visit did not affect eligibility. Emergency department visits were not included. Influenza vaccination status was combined from electronic medical record (EMR) data and self-report.

### Specimen collection

All patients except infants (<2 y) were sampled by two polyester swabs (Remel), one each on the nasal and oropharyngeal mucosa; infants were sampled by nasal swabs only. The swabs were combined in one cryovial containing viral transport medium, stored refrigerated, and delivered to the UPMC Clinical Virology Laboratory within 72 hours. Specimens were stored in a lysis buffer and aliquotted for nucleic acid isolation and detection of influenza virus using CDC’s SRT-PCR test and a MRT-PCR test using the eSensor XT-8 instrument and respiratory viral panel from GenMark Diagnostics. All SRT-PCR influenza positive specimens (n = 335) and a random sample of SRT-PCR influenza negative specimens (n = 596) were analyzed with MRT-PCR for a total of 935 of the 1171 specimens from the parent study. Four additional influenza cases were identified by MRT-PCR.

### Nucleic acid extraction and RT-PCR

Isolation of viral nucleic acid from control material and patient specimens was performed using an EasyMag automated extractor (bioMerieux, Durham, NC) as previously described [5]. Previously published, virus-specific primer and probe nucleotide sequences were used for detection of influenza A and B virus RNA [5] using the ABI 7500 Real-Time PCR Instrument (Applied Biosystems, Foster City, CA).

The eSensor RVP MRT-PCR assay (GenMark Diagnostics) is currently approved for clinical use in Europe. It has the same methodological characteristics but a broader range of viral analytes than the U.S. FDA-approved version. This panel includes adenovirus (ADNO) groups B, C, and E; coronaviruses (CoV) 229E, HKU1, OC43, and NL63; seasonal influenza A virus (including H1N1 and H3N2 subtype determination); influenza B virus; hMPV, PIV types 1, 2, 3, and 4; RSV types A and B; and HRV. The Genmark assay panel does not include enteroviruses, and in our laboratory, does not detect several major enteroviruses, i.e., enterovirus 68, enterovirus 71, and Coxsackie virus A9 (unpublished results).

Nucleic acids were extracted as for the SRT-PCR assay, with the addition of 10 μl of bacteriophage MS2 internal control (included in the eSensor RVP kit) to each specimen immediately prior to extraction. Specimens were tested by the eSensor XT-8 instrument according to the manufacturer's instructions and published protocols [6].

### Demographic and other variables

Participants completed surveys at enrollment from which age, race, personal smoking status and household smoking (someone in the household smokes), household composition, asthma diagnosis, exercise, influenza vaccination status, symptoms of ARI, self-reported overall health before ARI, subjective social status using a 10-point scale comparing one’s overall life situation with others, and self-reported severity of illness on day of enrollment, measured using a 100-point visual analog scale (VAS) were determined. Body Mass Index (BMI) was calculated from self-reported height and weight. Severity of illness, time to recovery, and loss of productivity were assessed on the follow-up survey completed at least 7 days post enrollment. Study data were collected and managed using REDCap electronic data capture tools [7].

### Statistical analyses

Similar viruses were combined and only single viruses or virus groups that were detected in more than 20 samples were used in the analyses. The final six groups were no virus detected, HRV, CoV, RSV, influenza virus type B, and influenza virus type A. Descriptive statistics are presented as means and standard deviations for continuous variables and percentages for discrete variables. Participants were divided into three age groups – children (6 months-17 years), young adults (18–49 years) and older adults (≥50 years). Bivariate multinomial regression models assessed the association of patient characteristics with the MRT-PCRs. The dependent variable was the virus group and the independent variables were the participants’ personal characteristics. Logistic regression models assessed the association of baseline demographic and clinical characteristics with virus type (single virus infection vs. co-detection). For discrete outcome measures with more than two levels, multinomial logistic regression models were used. One way analysis of variance was used for continuous outcomes. For time-to-event outcomes, Kaplan-Meier curves estimated the cumulative proportion of normal activity. Log rank tests were used to test for differences in the cumulative proportions among the virus groups. Post-hoc pairwise comparisons were made with a Bonferroni correction (p value < 0.05/15 indicating statistical significance). Analyses were conducted using SAS version 9.2 (SAS Institute, Inc., Cary NC).

## Results

### Relationship of viruses to demographic characteristics and symptoms

Among the 935 ARI patients sampled over the 17-week study period, all completed the enrollment questionnaire and 83.3% completed a follow-up survey.

**Figure 1:**
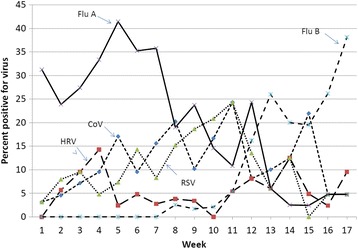
**Temporal epidemiology of virus distribution.**

The distributions of viral infections varied by week (*P* < 0.001), and are shown in Figure 1. Overall, HRV peaked in December with a second peak in March; influenza A peaked in January; CoV and RSV in February; and influenza B was not evident until late in the season and peaked in April. The overall cumulative distribution of return to usual activities was 1.5% by 1 day, 4.6% by 3 days, 10.9% by 5 days, 17% by 6 days, 29.3% by 7 days, 45.1% by 8 days and 100% by 9 days (data not shown).

Table 1 shows the demographic and illness characteristics of patients with five single virus groups and no virus detected. Hispanic ethnicity, vaccination status, and attendance at school outside the home varied significantly by virus category. For example, influenza vaccination was more often reported among those presenting with CoV, HRV and RSV than among those with either influenza A or B; 42% of those <18 years old with influenza A or B attended school outside the home compared with 26.3% of those with no virus detected. Fever and fatigue were more prominent with influenza detection and wheezing was less common with CoV. Use of influenza antiviral medicines varied significantly by virus category; 59% of those with influenza A or B were prescribed an antiviral medication compared with 14.8% of those with other viral infections or 26.2% of those with no viral infection. Temperature at enrollment was higher among those with influenza than those with ARI of other etiology, and baseline severity of illness was greater (lower score) among those with influenza. Days from onset to enrollment (seeking outpatient medical treatment) were greater among those with ARI with no virus detected than those with a viral infection.

**Table 1:** **Demographics and clinical characteristics by virus type in 2012-2013**

		**Virus type**												
	**All (N = 819)** ^**a**^	**CoV (N = 111)**		**HRV (N = 48)**		**Influenza A, H3 (N = 211)**		**Influenza B (N = 60)**		**RSV (N = 102)**		**No Virus (N = 287)**		***p*** **value**
**Variable**	**N**	**n**	**%**	**n**	**%**	**n**	**%**	**n**	**%**	**n**	**%**	**n**	**%**	
*Baseline Demographics and Health Characteristics (categorical responses)*														
Race														0.32
White	703	94	13.4	40	5.7	191	27.2	51	7.2	89	12.7	238	33.8	
Black	71	11	15.5	5	7	10	14.1	3	4.2	7	9.9	35	49.3	
Other	37	5	13.5	3	8.1	9	24.3	3	8.1	6	16.2	11	29.7	
Sex														0.14
Male	304	33	10.9	21	6.9	90	29.6	24	7.9	40	13.1	96	31.6	
Female	514	78	15.2	27	5.2	121	23.5	36	7	62	12.1	190	37.0	
Hispanic														**0.001**
No	799	110	13.8	48	6	209	26.2	52	6.5	101	13	278	34.9	
Yes	17	1	5.9	0	0	2	11.8	8	47	1	5.9	5	29.4	
Age group														0.10
6 mos. – 17 years	222	32	14.4	14	6.3	59	26.6	16	7.2	38	17.1	63	28.4	
18 – 49 years	346	54	15.6	19	5.5	86	24.8	26	7.5	30	8.7	131	37.9	
≥50 years	250	25	10	15	6	66	26.4	18	7.2	34	13.6	92	36.8	
Household members ≥ 18														0.41
1	154	26	16.9	8	5.2	35	22.7	7	4.6	17	11	61	39.6	
2	454	58	12.8	30	6.6	117	25.8	36	7.9	65	14.3	148	32.6	
≥3	210	27	12.8	10	4.8	59	28.1	17	8.1	20	9.5	77	36.7	
Household members < 18														0.26
0	369	51	13.8	21	5.7	102	27.6	23	6.2	43	11.7	129	35.0	
1	167	21	12.6	10	6	36	21.5	9	5.4	29	17.4	62	37.1	
2	166	17	10.2	13	7.8	45	27.1	15	9	20	12.1	56	33.7	
≥3	115	22	19.1	4	3.5	28	24.4	13	11.3	10	8.7	38	33.0	
Social status														0.26
1-4	103	15	14.6	5	4.8	28	27.2	7	6.8	10	9.7	38	36.9	
5	150	24	16	7	4.7	30	20	11	7.3	12	8	66	44.0	
6	122	16	13.1	13	10.7	31	25.4	7	5.7	17	13.9	38	31.2	
7 - 9	218	24	11	8	3.7	63	28.9	18	8.2	24	11	81	37.2	
Smoking status														0.11
Smoker	108	18	16.7	7	6.5	27	25	6	5.5	4	3.7	46	42.6	
Non-smoker	488	61	12.5	27	5.5	125	25.6	38	7.8	60	12	177	36.3	
Household smoking														0.18
Yes	167	20	12	10	6	42	25.1	7	4.2	17	10.2	71	42.5	
No	649	91	14	38	5.9	167	25.7	53	8.2	85	13.1	215	33.1	
Asthma status														0.26
Yes	183	18	9.8	13	7.1	45	24.6	15	8.2	18	9.8	74	40.5	
No	630	92	14.6	35	5.5	164	26	44	7	83	13.2	212	33.7	
Influenza vaccination status														**0.05**
Yes	403	59	14.6	30	7.4	88	21.9	27	6.7	56	13.9	143	35.5	
No	409	51	12.5	18	4.4	121	29.6	33	8.1	44	10.7	142	34.7	
Self-reported health status														0.32
Fair/poor	49	6	12.2	3	6.1	9	18.4	1	2.1	5	10.2	25	51.0	
Good	226	31	13.7	10	4.4	55	24.4	19	8.4	26	11.5	85	37.6	
Very Good	323	45	13.9	19	5.9	86	26.6	31	9.6	42	13	100	31.0	
Excellent	219	29	13.2	16	7.3	61	27.9	9	4.1	28	12.8	76	34.7	
Current employment (age ≥18 years)														0.18
No	145	18	12.4	7	4.8	34	23.4	7	4.8	22	15.2	57	39.3	
Yes	351	48	13.7	23	6.6	97	27.6	31	8.8	32	9.1	120	34.2	
Attends school outside home (age <18 years)														**0.02**
No	34	6	17.6	4	11.7	4	11.7	1	2.9	11	32.3	8	23.5	
Yes	148	18	12.2	9	6.1	49	33.1	13	8.8	20	13.5	39	26.3	
*Symptoms of ARI and Antiviral Use (categorical responses)*														
Fever														**<.001**
Yes	479	61	12.7	19	4**	164	34.2*	45	9.4	53	11.1^‡^	137	28.6	
No	339	50	14.7	29	8.5	47	13.9	15	4.4	49	4.5	149	44	
Fatigue														**<.001**
Yes	634	87	13.7	30	4.7**	182	28.7	54	8.5	74	11.7	207	32.7	
No	184	24	13	18	9.8	29	15.8	6	3.3	28	15.2	79	42.9	
Wheezing														**0.01**
Yes	297	25	8.4	18	6.1	89	30*	23	7.7	45	15.1	97	32.7	
No	521	86	16.5	30	5.8	122	23.4	37	7.1	57	10.9	189	36.3	
Sore throat														0.11
Yes	589	84	14.3	37	6.3	164	27.8	39	6.6	71	12.1	194	32.9	
No	229	27	11.8	11	4.8	47	20.5	21	9.2	31	13.5	92	40.2	
Nasal congestion														0.12
Yes	711	101	14.2	45	6.3	183	25.7	51	7.2	93	13.1	238	33.5	
No	107	10	9.4	3	2.8	28	26.2	9	8.4	9	8.4	48	44.8	
Shortness of breath														0.52
Yes	352	49	13.9	19	5.4	90	25.6	33	9.4	43	12.2	118	33.5	
No	466	62	13.3	29	6.2	121	26	27	5.8	59	12.7	168	36	
Antiviral medication prescribed, (not including HRV) ^†^														**0.002**
No	564	83	14.7	-	-	151	26.8	47	8.3	80	14.2	203	36	
Yes	61	5	8.2	-	-	31	50.8	5	8.2	4	6.6	16	26.2	
*Baseline Demographics and Symptoms (continuous variables)*														
Variable	N	n	Mean (SD)	n	Mean (SD)	n	Mean (SD)	n	Mean (SD)	n	Mean (SD)	n	Mean (SD)	p value
Age	818	111	33.8 (21.4)	48	32.8 (21.2)	211	36.2 (22.3)	60	34.9 (20.6)	102	34.7 (20.6)	286	37.2 (21.3)	0.65
BMI	817	110	20.5 (14.2)	48	20.6 (14.0)	211	21 (14.3)	60	21.7 (13.9)	102	18.7 (15.2)	286	22.9 (13.4)	0.16
Temperature	559	72	98.7 (1.10)	26	98.6 (0.80)	161	99.6 (1.50)	44	99.8 (1.50)	62	98.9 (1.20)	194	98.9 (1.20)	**<0.001**
Days from onset to enrollment	818	111	3.23 (1.46)	48	3.35 (1.45)	211	2.82 (1.40)	60	3.38 (1.22)	102	3.55 (1.35)	286	3.71 (1.48)	**<0.001**
Severity of illness at enrollment (VAS), range = 1 - 100	818	111	62.2 (18.2)	48	64.9 (21.2)	211	55.7 (17.7)	60	56.3 (20.5)	102	61.3 (17.4)	286	61.3 (19.2)	**0.002**

^a^N reflects the total number of participants, reduced by the number of co-detections and viruses with <20 detections.

*Significantly different from CoV in post-hoc comparisons *P* ≤ 0.001; post-hoc comparisons are based on a Bonferroni correction for multiple comparison (*P* < .003).

**Significantly different from Influenza A and B in post hoc comparisons, *P* ≤ 0.001.

^‡^Significantly different from Influenza A in post hoc comparisons, *P* ≤ 0.001

^†^Antiviral medication was never prescribed for those with HRV, leading to cells with zeroes in them.

**Figure 2:**
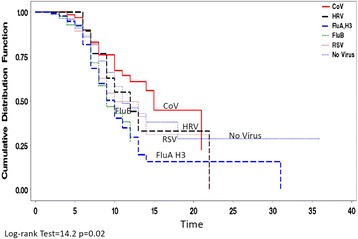
**Testing homogeneity of survival curves for time to return to normal activity over virus groups.**

Table 2 shows the outcomes for patients in the five single virus groups and those with no virus detected. Hours of work missed due to ARI varied significantly by virus group, appearing worse for influenza A and B. Self-reported time to return to normal activity varied by virus group (*P* = 0.02) and is depicted in Figure 2.

**Table 2:** **Outcomes of patients presenting with medically attended acute respiratory infections by viral etiology in 2012-2013**

	**Virus type**													
***Outcomes (categorical variables)***	**All (N = 684)** ^**a**^	**CoV (91)**		**HRV (43)**		**Influenza A, H3 (185)**		**Influenza B (52)**		**RSV (86)**		**No Virus (227)**		***p*** **value**
**Variable**	**N**	**n**	**%**	**n**	**%**	**n**	**%**	**n**	**%**	**n**	**%**	**n**	**%**	
Follow-up survey returned relative to enrollment date														**<0.001**
0 – 13 days	394	36	9.1	23	5.8	133	33.8	44	11.2	49	12.4	109	27.7	
≥14 days	288	55	19.1	20	6.9	51	17.7	8	2.8	37	12.9	117	40.6	
Ability to perform usual activity, range = (not at all) – 9 (able to perform)														0.08
Not at all (0 – 5)	74	9	10	4	9.5	18	9.7	8	15.4	11	12.9	24	10.8	
Somewhat (6 – 8)	232	23	25.6	15	35.7	80	43.3	22	42.3	26	30.6	66	29.6	
Able to perform (9)	371	58	64.4	23	54.8	87	47	22	42.3	48	56.5	133	59.6	
Household member missed work because of patient’s illness														0.40
No	563	74	81.3	38	88.4	149	80.5	40	76.9	68	79.1	194	85.8	
Yes	120	17	18.7	5	11.6	36	19.5	12	23.1	18	20.9	32	14.2	
Sleep quality, range = 0 (worst) – 9 (normal)														0.67
Worst (0 – 4)	52	8	9	4	9.5	10	5.4	2	3.8	9	10.6	19	8.5	
Mild (5 – 6)	101	15	16.8	6	14.3	26	14	7	13.5	11	12.9	36	16.1	
Moderate (7 – 8)	242	36	40.5	11	26.2	68	36.8	23	44.2	34	40	70	31.4	
Normal (9)	280	30	33.7	21	50	81	43.8	20	38.5	31	36.5	98	44	
Effect on productivity, range = 0 (no effect on my work) – 10 (completely prevented me from working for those age ≥18 years)														0.62
0 – 3	77	15	31.3	5	21.7	17	17.5	4	12.9	7	21.9	29	24.4	
4 – 7	153	16	33.3	10	43.5	41	42.3	16	51.6	15	46.9	55	46.2	
8 – 10	120	17	35.4	8	34.8	39	40.2	11	35.5	10	31.2	35	29.4	
***Outcomes (continuous variables)***														
**Variable**	**N**	**n**	**Mean (SD)**	**n**	**Mean (SD)**	**n**	**Mean (SD)**	**n**	**Mean (SD)**	**n**	**Mean (SD)**	**n**	**Mean (SD)**	***p*** **value**
For participants <18 years:														
Missed school days	147	18	2.4	9	2.1	49	2.9	13	2.9	20	1.8	38	2.5	0.47
			(1.9)		(2.0)		(2.4)		(1.9)		(1.3)		(2.1)	
Household member missed work days	118	16	2	5	3	35	3	12	2	18	2	32	2	0.19
			(1.1)		(2.0)		(2.9)		(1.9)		(1.8)		(1.3)	
Severity of illness at follow-up (VAS), range = 1-100	658	86	83 (15.1)	42	83 (16.0)	181	83 (14.4)	50	82 (13.0)	81	83 (14.7)	218	84 (15.7)	0.98
For participants ≥18 years:														
Number of hours of work missed due to ARI	351	48	14 (12.5)	23	16 (18.0)	97	22 (16.4)	31	22 (17.9)	32	11 (11.5)	120	12 (13.1)	**0.001**
***Multinomial Modeling: Odds Ratios for derived variables***		**CoV (n = 91)**		**HRV (n = 43)**		**Influenza A, H3 (n = 185)**		**Influenza B (n = 52)**		**RSV (n = 86)**		**No Virus (n = 227)**		
		**OR**		**OR**		**OR**		**OR**		**OR**		**OR**		***p*** **value**
**Variable**		**(95% CI)**		**(95% CI)**		**(95% CI)**		**(95% CI)**		**(95% CI)**		**(95% CI)**		
Ability to perform usual activities														0.08
Not at all (0–5) vs. Able to perform (9)		0.74		0.83		0.99		1.74		1.10		Referent		
		(0.39-1.41)		(0.33-2.08)		(0.59-1.65)		(0.84-3.58)		(0.59-2.02)				
Somewhat able (6–8) vs. Able to perform (9)		0.63		1.03		1.45		1.58		0.86		Referent		
		(0.4-0.98)		(0.59-1.82)		(1.06-2.00)		(0.94-2.66)		(0.55-1.33)				
Household member missed work (days)		1.39		0.80		1.47		1.82		1.61		Referent		0.40
		(0.73-2.66)		(0.29-2.18)		(0.87-2.47)		(0.86-3.83)		(0.85-3.04)				
Sleep Quality 0 (worst quality, unable to sleep) – 9 (pre-illness quality sleep)														0.68
Worst vs. Normal		1.47		1.05		0.68		0.55		1.60		Referent		
		(0.70-3.08)		(0.41-2.72)		(0.35-1.31)		(0.16-1.92)		(0.79-3.27)				
Mild vs. Normal		1.40		0.80		0.90		0.98		0.99		Referent		
		(0.79-2.49)		(0.36-1.76)		(0.57-1.41)		(0.46-2.08)		(0.53-1.86)				
Moderate vs. Normal		1.36		0.59		0.95		1.30		1.24		Referent		
		(0.87-2.12)		(0.32-1.11)		(0.68-1.33)		(0.76-2.22)		(0.79-1.94)				
Effect on productivity														0.64
Worst vs. No effect		0.69		0.97		1.40		1.67		0.87		Referent		
		(0.35-1.35)		(0.37-2.59)		(0.78-2.51)		(0.62-4.55)		(0.37-2.06)				
Moderate vs. No effect		0.51		0.96		1.15		1.91		1.02		Referent		
		(0.26-1.00)		(0.37-2.45)		(0.65-2.05)		(0.73-4.98)		(0.46-2.30)				
***Multinomial Modeling: Hazards Ratio***		**HR**		**HR**		**HR**		**HR**		**HR**		**HR**		***p value***
**Variable**		**(95% CI)**		**(95% CI)**		**(95% CI)**		**(95% CI)**		**(95% CI)**		**(95% CI)**		
Time to return to normal activity (days)		0.74		1.06		**1.65**		1.48		1.22		Referent		**0.02**
		(0.45-1.22)		(0.53-2.09)		**(1.13-2.39)**		(0.89-2.46)		(0.73-2.05)				

OR= Odds Ratio; CI = Confidence Interval; SD = Standard Deviation; HR = Hazards ratio.

^a^N reflects the total number of participants, reduced by the number of co-detections, viruses with <20 detections, and participants without follow-up surveys.

### Distribution of viruses and co-detections

Table 3 shows the distribution of single virus infections and co-detections, with the numbers for co-detections representing viruses detected, not numbers of patients. Single virus infections were detected among 563 (60.2%) patients, co-detections were found among 85 (9.1%) patients and 287 (30.7%) were negative for all tested viruses. The viruses most frequently co-detected were CoV (49 times), RSV (39 times), and influenza A (39 times). The distributions varied by age. For children, the percentages of single virus, multiple viruses, and no virus detected were 63%, 14%, and 23%, respectively; whereas for young adults, the percentages were 58%, 8%, and 34% and for older adults the percentages were 61%, 5%, and 32%, respectively (*P* < 0.001 for overall distributions; data not shown).

**Table 3:** **Distribution of Viruses from multiplex viral panel Among 935 outpatients with medically attended acute respiratory infections in 2012–13**

**Virus**	All samples (N = 935) tested by multiplex RT-PCR,* n (%)	
**Singly occurring viruses by type**		
Coronaviruses (CVOC43, n = 72; CVNL63, n = 33; CV229E, n = 4; CVKHU1, n = 2)	111 (11.9)	
HRV	48 (5.1)	
Influenza type A	213 (22.8)	
Influenza type B	60 (6.4)	
RSV (A, n = 85 and B, n = 17)	102 (10.9)	
Other single viruses (ADNOE, n = 1; HMPV, n = 8; PIV1, n = 2; PIV2, n = 9; PIV3, n = 8; PIV4, n = 1)	29 (3.1)	
**Single viruses total**	563 (60.2)	
**Co-detections**	85 (9.1)	
**No viruses**	287 (30.7)	
**Co-detected viruses**		
Virus	Co-detected viruses	n^†^
ADNO	Dual – CoV, HRV, RSV, Influenza B	5
	Triple – CoV/Influenza A, HMPV/RSV	2
CoV	Dual – ADNO, CoV, HRV, Influenza B, HMPV, PIV, RSV	45
	Triple – Influenza A/HRV, Influenza A/ADNO, HRV/RSV	4
HMPV	Dual - CoV, Influenza A, RSV	4
	Triple – RSV/ADNO	1
HRV	Dual – ADNO, CoV, Influenza B, RSV	10
	Triple – Influenza A/CoV, RSV/CoV	3
Influenza A	Dual – CoV, HRV, HMPV, RSV, PIV	36
	Triple – ADNO/CoV, HRV/CoV	2
Influenza B	Dual – CoV, ADNO, HRV, Influenza A	9
PIV	Dual – CoV, RSV, Influenza A	6
RSV	Dual – ADNO, CoV, HRV, Influenza A, HMPV, PIV	36
	Triple – HRV/CoV, HMPV/ADNO	3

*MRT-PCR = multiplex reverse transcriptase polymerase chain reaction; ADNO = Adenovirus.

CoV = Coronavirus; HMPV = Human Metapneumovirus; HRV = Human Rhinovirus.

RSV = Respiratory Syncytial Virus; one dual H1N1/H3N2 detection occurred.

^†^Represents occurrences of viruses, not individual participants.

Compared with single infections, co-detections were more common in children than older adults (Table 4) and less frequent in households without children. Co-detections were less common if sore throat was present, but did not vary by other symptoms. Average body mass index was below normal among those with co-detections and more likely to be low among this group compared with those with single infections.

**Table 4:** **Baseline comparisons using unadjusted odds ratios, between single viral infections (n = 563) and co-detections (n = 85) in 2012-2013**

**Variable**	**Single infection %**	**Co-detection %**	**Unadjusted OR (95% CI)**	***P*** **-value**
*Categorical variables*				
Race				0.180
White	87.7	12.3	0.61 (0.24-1.53)	
Black	79.6	20.4	1.11 (0.36-3.43)	
Other	81.2	18.8	Referent	
Sex				0.780
Male	87.3	12.7	Referent	
Female	86.6	13.4	1.07 (0.67-1.71)	
Hispanic				0.940
No	86.9	13.1	Referent	
Yes	87.5	12.5	0.95 (0.21-4.23)	
Age group				**0.010**
6 mo-17 years	81.6	18.4	2.51 (1.33-4.72)	
18-49 years	87.8	12.2	1.55 (0.81-2.96)	
≥50 years	91.8	8.2	Referent	
Household members ≥18				0.690
1	89.2	10.8	0.82 (0.39-1.74)	
2	86.1	13.9	1.09 (0.63-1.88)	
≥3	87.1	12.9	Referent	
Household member <18				**0.003**
0	92.0	8.0	0.45 (0.23-0.90)	
1	86.6	13.4	0.80 (0.38-1.68)	
2	79.4	20.6	1.34 (0.69-2.62)	
≥3	83.8	16.2	Referent	
Subjective social status, range = 0-9				0.910
1-4	87.2	12.8	1.25 (0.55-2.88)	
5	90.5	9.5	0.89 (0.38-2.09)	
6	89.8	10.2	0.97 (0.43-2.21)	
≥7	89.5	10.5	Referent	
Smoking status				0.260
Smoker	85.9	14.1	1.52 (0.73-3.13)	
Non-smoker	90.2	9.8	Referent	
Household smoking				0.910
Yes	86.7	13.3	1.03 (0.58-1.86)	
No	87.1	12.9	Referent	
Asthma Status				0.600
Yes	85.4	14.6	1.16 (0.67-1.99)	
No	87.1	12.9	Referent	
Influenza vaccination status				0.220
Yes	88.5	11.5	Referent	
No	85.1	14.9	1.34 (0.84-2.12)	
Self-reported health status				0.090
Fair/Poor	89.7	10.3	0.54 (0.15-1.87)	
Good	86.0	14.0	0.76 (0.43-1.34)	
Very good	90.4	9.6	0.49 (0.28-0.86)	
Excellent	82.3	17.7	Referent	
Ability to perform usual activities				0.190
Not at all (0–5)	84.8	15.2	2.21 (0.85-5.81)	
Somewhat (6–8)	87.9	12.1	1.71 (0.64-4.57)	
Able to perform (9)	92.5	7.5	Referent	
Current employment				0.170
Yes	89.0	11.0	Referent	
No	93.8	6.2	0.53 (0.21-1.32)	
Child attends school outside home (for those <18 years old)				0.090
Yes	85.1	14.9	Referent	
No	73.8	26.2	2.02 (0.88-4.67)	
Anti-viral medication prescribed				0.840
Yes	86.8	13.2	Referent	
No	87.7	12.3	0.92 (0.40-2.13)	
Fever				0.660
Yes	86.4	13.6	1.11 (0.69-1.80)	
No	87.7	12.3	Referent	
Fatigue				0.370
Yes	87.5	12.5	0.78 (0.46-1.33)	
No	84.5	15.5	Referent	
Wheezing				0.350
Yes	85.3	14.7	1.24 (0.78-1.98)	
No	87.8	12.2	Referent	
Sore throat				**0.010**
Yes	89.2	10.8	0.51 (0.32-0.82)	
No	81.0	19.0	Referent	
Nasal congestion				0.640
Yes	87.1	12.9	0.85 (0.43-1.68)	
No	85.1	14.9	Referent	
Shortness of breath				0.600
Yes	87.7	12.3	0.88 (0.56-1.40)	
No	86.3	13.7	Referent	
*Continuous variables*				
Age, years (units = 5)	34.6 (22.8)	26.3 (20.9)	0.92 (0.87-0.97)	**0.002**
BMI (units = 2)	20.2 (14.4)	15.3 (14.5)	0.95 (0.92-0.98)	**0.004**
Temperature (°F)	99.2 (1.4)	99.3 (1.1)	1.03 (0.85-1.26)	0.740
Severity of illness at enrollment (VAS), range = 1–100 (units = 5)	59.2 (18.6)	56.7 (19.1)	0.96 (0.91-1.02)	0.240
Number of days between onset and enrollment	3.2 (1.4)	3.3 (1.3)	1.05 (0.90-1.24)	0.520
Number of days between enrollment and return of follow-up survey (units = 5)	14.8 (10.9)	15.9 (10.6)	1.04 (0.94-1.16)	0.440

Ability to perform usual activities, return to normal activities, household members having to miss work, sleep quality, productivity, severity of illness, hours of work missed, and time to return to normal activities did not differ between those with single infections and those with co-detected viruses (Table 5). However, those <18 years of age with co-detections missed fewer days of school (2 vs. 1.1, *P* = 0.04) and all with co-detections reported fewer days of work missed by household members to care for the ill patient (3 vs. 2, *P* = 0.03).

**Table 5:** **Follow-up comparisons between single viral infections (n = 483) and co-detections (n = 68), 2012-2013**

	**Virus type**			
	**Single infection**	**Co-detection**	**Unadjusted**	***P*** **value**
	**N = 483**	**N = 68**	**OR (95% CI)**	
**Variable**	**%**	**%**		
Ability to perform usual activities				0.450
Not at all (0–5)	10.8	6.0	0.74 (0.43-1.26)	
Somewhat (6–8)	36.1	40.3	1.05 (0.80-1.37)	
Able to perform (9)	53.1	53.7	Referent	
Return to normal activity				0.570
Not yet return to normal activity	40.6	47.4	1.32 (0.50-3.46)	
Never stopped normal activities	59.4	52.6	Referent	
Household member missed work				0.100
No	80.7	72.1	Referent	
Yes	19.3	27.9	1.63 (0.91-2.89)	
Sleep quality, range = 0-9				0.470
Worst (0–4)	7.1	7.5	0.97 (0.59-1.61)	
Mild (5–6)	13.6	17.9	1.09 (0.76-1.56)	
Moderate (7–8)	37.8	28.4	0.82 (0.61-1.11)	
Normal (9)	41.5	46.2	Referent	
Effect on productivity (age ≥18 years)				0.130
No effect (0–3)	20.2	13.8	Referent	
Moderate (4–7)	43.8	31.0	1.02 (0.84-2.67)	
Worst (8–10)	36.0	55.2	1.50 (0.55-1.88)	
	**Mean (SD)**	**Mean (SD)**		***P*** **value**
Missed school days due to ARI	2.0 (2.1)	1.1 (1.3)		**0.040**
Missed work days of household member	3.0 (2.2)	2.0 (0.9)		**0.030**
Severity of illness at follow-up (VAS), range = 1-100	83.0 (14.6)	86.0 (15)		0.140
Hours of work missed due to ARI among ≥18 years-old	18.0 (15.8)	18.0 (12.1)		0.930

## Discussion

This study, similar to a study conducted in a previous influenza season [1], examined the distribution of 18 viruses among individuals seeking outpatient care for ARI characterized by presence of cough. The 2012–2013 influenza season was markedly different from the previous one. Although both seasons were characterized by a preponderance of influenza A/H1N1pdm09, in 2011–2012, influenza viruses began circulating in this region at low levels in December, but did not peak until the spring of 2012, with few influenza B virus detections. Whereas, in 2012–2013, influenza A began circulating in December, began to wane in late February as a smaller wave of influenza B began appearing in late February and early March. Concurrently, HRV peaked in December and in March, earlier than the previous year when it peaked in April; CoV was active from late December through March and RSV was active in February.

Significant differences between viral infections and symptom variables were observed with fever and fatigue being more prominent with influenza and wheezing less common with CoV. Such findings are consistent with the literature showing that fever is a common symptom for both HMPV and influenza [8]. Enrollees with influenza seem to have been sicker, as they more frequently reported fever, feeling worse at baseline, seeking treatment sooner and, among adults, missing more work time.

We found that co-detections occurred in 9% of ARI cases. CoV, RSV and influenza A were the most common co-detections, whereas, in the previous year, co-detections were most frequently caused by CoV, HRV and influenza A [1] and in a longer, year round study, HRV was the most frequent co-infecting virus [9]. We also observed some triple co-detections involving ADNO, CoV, HMPV, RSV, influenza A, and HRV. Importantly, we found that co-detections were more common in children, and were less frequent in households without children. The association of virus co-detections with younger age fits with the known enhanced susceptibility of children to these single infections.

The clinical impact of co-detections is debatable. In the literature, dual infections have been linked to more severe clinical outcomes compared with single virus infections. These studies have included primarily children <5 years, and adults with co-morbidities [10], and children presenting to emergency departments [11], or resulted from multiple influenza virus infections [12]. However, in our population of adults and children, we found that co-detections were significantly less common if sore throat was present and did not vary by other symptoms. Moreover, compared with individuals with single viral infections, those with co-detections missed significantly fewer days of school (1.1 vs. 2 days) or work (2 vs. 3 days). A recent study of children attending day care in which no demographic or household variables were related to co-infections, found that fever was less likely with co-infections [13]. Hence, some data emerging from our study and others using newly available, highly sensitive and specific assays for multiple virus infections have not found a relationship with more severe illness whereas others have. Indeed, our data suggest that multiple respiratory virus infections are associated with less severe clinical symptoms particularly fever, and are less detrimental to the patients’ quality of life. The co-detections may represent a commensal situation in which the second virus has no effect on the predominant infection but is found with these sensitive tests. The contrast in results between studies on co-detections finding a less versus more severe clinical course may be related to population, test characteristics, time of year, and duration of testing period. Further studies are needed to define underlying viral and host immune mechanisms and outcomes of multiple respiratory virus infections.

The sensitivity and specificity of the MRT-PCR compared with SRT-PCR were similar for both years. The sensitivity was 91.1% and specificity was 98.2% in 2011–2012 [1] and the sensitivity was 96% and specificity was 99.8% for influenza A in 2012–2013 [14]. In 2012–13, most of the discordant results were weak positives on SRT-PCR and the discordant results included five specimens in which the MRT-PCR was positive for another virus.

### Strengths and limitations

This study offers data on viral infections associated with outpatient ARI in the US during the winter influenza season, detected using the eSensor 18 virus panel currently available in Europe. This panel of viruses includes four CoVs and four PIVs that are not part of the currently (2015) FDA-cleared format. Study limitations include the inability to test an inclusive sample of all of the ARI specimens with the MRT-PCR and the fact that health-seeking behavior during illness is associated with a history of influenza vaccination. The sample size of detected viruses other than influenza, HRV, RSV and coronaviruses were too small for sub-analyses. However, the sample size overall was sufficient to allow confidence in the relationships between characteristics of ARI cases and the viruses associated with them during this time period. Additionally, the viral panel does not contain bacteria, mycoplasma, and all possible respiratory viruses. Sampling took place during the influenza season and may have missed peak seasons for other virus circulation. The study is strengthened by the similarity of methods used in a previous influenza season and allows for comparison of viral activity in two seasons with different influenza epidemiology.

## Conclusions

In this study using multiplex RT-PCR, 69% of outpatient medically attended ARI during the 2012–2013 influenza season were associated with a viral etiology. The timing and distribution of viral infections differed from a previous influenza season. Co-infections were infrequent but varied by demographic and household characteristics.

### Ethical approval

This study was approved by the University of Pittsburgh Institutional Review Board.
